# In-depth comparison of cell-based methodological approaches to determine drug susceptibility of visceral *Leishmania* isolates

**DOI:** 10.1371/journal.pntd.0007885

**Published:** 2019-12-02

**Authors:** Sarah Hendrickx, Lieselotte Van Bockstal, Guy Caljon, Louis Maes

**Affiliations:** Laboratory of Microbiology, Parasitology and Hygiene (LMPH), University of Antwerp, Antwerp, Belgium; University of Tokyo, JAPAN

## Abstract

Monitoring the drug susceptibility of *Leishmania* isolates still largely relies on standard *in vitro* cell-based susceptibility assays using (patient-isolated) promastigotes for infection. Although this assay is widely used, no fully standardized/harmonized protocol is yet available hence resulting in the application of a wide variety of host cells (primary cells and cell lines), different drug exposure times, detection methods and endpoint criteria. Advocacy for standardization to decrease inter-laboratory variation and improve interpretation of results has already repeatedly been made, unfortunately still with unsatisfactory progress. As a logical next step, it would be useful to reach at least some agreement on the type of host cell and basic experimental design for routine amastigote susceptibility determination. The present laboratory study using different *L*. *infantum* strains as a model for visceral leishmaniasis species compared primary cells (mouse peritoneal exudate (PEC), mouse bone marrow derived macrophages and human peripheral blood monocyte derived macrophages) and commercially available cell lines (THP-1, J774, RAW) for either their susceptibility to infection, their role in supporting intracellular amastigote multiplication and overall feasibility/accessibility of experimental assay protocol. The major findings were that primary cells are better than cell lines in supporting infection and intracellular parasite multiplication, with PECs to be preferred for technical reasons. Cell lines require drug exposure of >96h with THP-1 to be preferred but subject to a variable response to PMA stimulation. The fast dividing J774 and RAW cells out-compete parasite-infected cells precluding proper assay read-out. Some findings could possibly also be applicable to cutaneous *Leishmania* strains, but this still needs cross-checking. Besides inherent limitations in a clinical setting, susceptibility testing of clinical isolates may remain problematic because of the reliance on patient-derived promastigotes which may exhibit variable degrees of metacyclogenesis and infectivity.

## Author summary

Leishmaniasis is a neglected tropical disease caused by parasites belonging to the genus of *Leishmania* and transmitted by the bite of infected female sand flies. Concerns about the effective control of the disease are rising in view of the increasing number of treatment failures that may be related to drug resistance. Monitoring of drug susceptibility in the field should become an essential asset, however, there is still insufficient harmonization in the laboratory assays. This study focused on the standard intracellular amastigote susceptibility assay and compared protocol variables, such as type of macrophage host cell (primary versus cell lines), multiplicity of infection and duration of drug exposure. Primary cells perform best with little difference between cells derived from Swiss mice or BALB/c mice. From a practical point of view, mouse peritoneal exudate cells can be recommended. If mice would not be available, THP-1 cells are the best alternative. For field strains, metacyclic promastigotes should be used at a multiplicity of infection of 10–15 parasites per cell with drug exposure starting at 24h post-infection and continued for 120h. Unfortunately, susceptibility testing of clinical isolates will remain problematic because of the reliance on promastigotes which may exhibit variable degrees of metacyclogenesis and infectivity. Opting for cell-based assays may be complicated by the fact that dedicated laboratory infrastructure may sometimes be lacking in disease-endemic countries.

## Introduction

**Fig 1:**
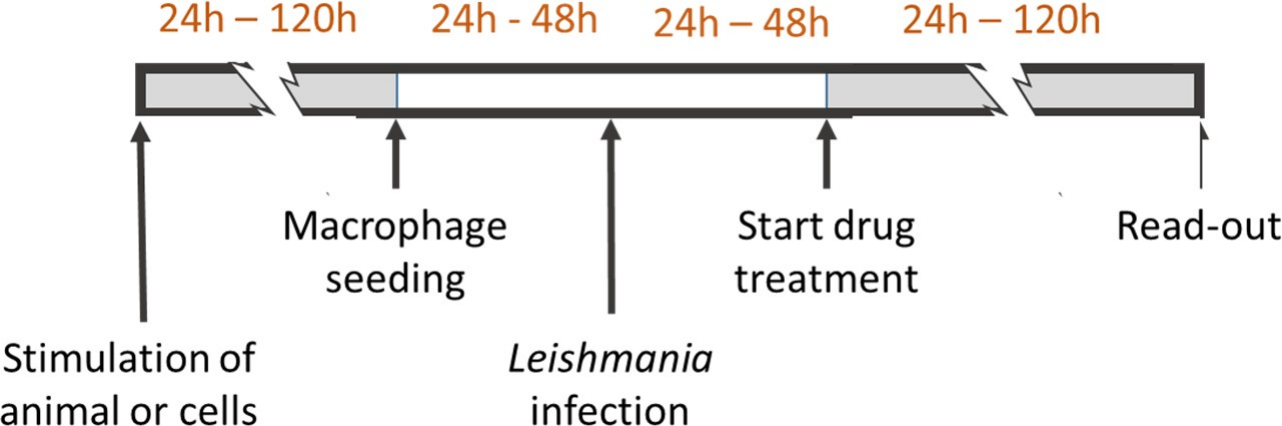
Variability in the standard intracellular susceptibility protocol. Divergences in design related to the application of different host cells, different host and/or cell stimuli and different drug exposure times.

Despite the ongoing search for specific molecular resistance markers [1–3], the approach to evaluate drug resistance in *Leishmania* still heavily relies on *in vitro* drug-susceptibility assays. Given the well-known stage-dependent susceptibility differences that can be observed for most drugs [4, 5], intracellular amastigote assays may be regarded as the gold standard [5], although assays on extracellular promastigotes or axenic amastigotes can be informative as well with the advantage that they are much more accessible and less time-consuming. Intracellular assays require a suitable host cell and several types have already been described and compared in literature, covering the THP-1, RAW, J774 and U937 cell lines, and various types of primary macrophages [6–15]. For example, monocyte-derived cell lines require cell stimulation where the different use concentration of phorbol-12-myristate-13-acetate (PMA), retinoic acid or 1,25-dihydroxy-vitamin D3 during variable stimulation periods precludes direct comparison of results [16, 17]. Although most protocols are basically constructed in a similar way, the abundance of marginally different designs (Fig 1) nevertheless complicates drug susceptibility interpretation and inter-laboratory comparison. Despite several pleas for standardization [18–20] and literature on comparative evaluation of several cell types [14, 20, 21], different models are still widely used hence requiring further refinement of the proposed experimental methodology.

Recognizing the necessity to harmonize *Leishmania* drug evaluation assays and establish standardized methods to monitor drug resistance, as was done for malaria (WWARN: WorldWide Antimalarial resistance Network; www.wwarn.org) [18–20], the present study aimed to contribute to a pragmatic and straightforward approach to determine the drug susceptibility of (clinical) *Leishmania* isolates in laboratory conditions, hereby supporting a next step towards a more harmonized operating procedure that could find some application in the clinical setting. Strains of *L*. *infantum* were used and specific focus was given to the suitability of different types of primary macrophages and cell lines as permissive host cell, receptivity to infection, capacity to support intracellular multiplication and the susceptibility outcome of the most frequently used antileishmanial reference drugs.

## Materials and methods

### Media, reagents and chemicals

M199 medium, DMEM-medium, RPMI-1640, MEM non-essential amino acids, L-glutamine, penicillin-streptomycin (P/S) solution, fetal bovine serum (FBS), sodium pyruvate, phosphate buffered saline (PBS) and hemin were purchased from Invitrogen (Ghent, Belgium). Phorbol 12-myristate 13-acetate (PMA), trivalent antimony (Sb^III^: potassium antimonyl tartrate), EDTA, HEPES, potato starch, Giemsa, adenine, paromomycin (PMM) and Histopaque-1077 were purchased from Sigma Aldrich (Diegem, Belgium). Miltefosine (MIL) was available from Carbosynth (Berkshire, UK), amphotericin B deoxycholate (AmB: Fungizone Bristol-Myers Squibb (Brussels, Belgium), sodium-stibogluconate (Sb^V^; SSG) from Calbiochem (San Diego, US) and 6-biopterine from Schrickx Labs (Bauma, Switzerland).

### Preparation of drug stock solutions

Drug stock solutions were prepared as described previously [22]. The Sb^V^ stock solution (1 mg Sb^V^ eq /ml) was prepared by dissolving 31.9 mg SSG in 10 ml water or PBS by stirring at 37°C for 1 hour until a clear solution was obtained which was divided into small aliquots (1 ml) kept at -20°C for maximally 3 months. To prepare a Sb^III^ stock solution (1 mg Sb^III^ eq /ml), 27.7 mg Sb^III^ tartrate was dissolved in 10 ml water or PBS by stirring until a clear solution was obtained. Similar as for Sb^V^, the suspension was divided into small aliquots and kept at -20°C for maximally 3 months. To obtain the AmB 20 mM stock solution, 41 mg Fungizone powder was dissolved in 1 ml DMSO 100%. Further dilutions were made in water and immediately used because of limited stability. A 20 mM MIL stock solution was prepared by dissolving 81.5 mg MIL in 10 ml water or PBS by stirring until a clear solution was obtained. The MIL stock solution can be kept at 4°C for 3 months. Finally, PMM stock solutions (20 mM) were made by dissolving 142.74 mg PMM-sulfate in 10 ml water or PBS. The stock solution was kept at 4°C for maximally 3 months.

### Ethics

The use of laboratory rodents was carried out in strict accordance to all mandatory guidelines (EU directives, including the Revised Directive 2010/63/EU on the Protection of Animals used for Scientific Purposes that came into force on 01/01/2013, and the declaration of Helsinki in its latest version) and was approved by the ethical committee of the University of Antwerp, Belgium (UA-ECD 2016–54 (02-09-2016).

### Laboratory animals

Female Swiss and BALB/c mice (body weight 20 g) for the collection of primary peritoneal and bone marrow derived macrophages were purchased from Janvier (Le Genest Saint Isle, France). Food for laboratory rodents (Carfil, Arendonk, Belgium) and drinking water were available *ad libitum*. Animals were kept in quarantine for at least 5 days before the collection of macrophages.

### *Leishmania* parasites, cultures and infection

All experiments were carried out using the *Leishmania infantum* laboratory reference strain (MHOM/MA/67/ITMAP263) and three *L*. *infantum* clinical isolates with a defined drug susceptibility background to the standard reference drugs antimony (Sb^V^ and Sb^III^), miltefosine (MIL), paromomycin (PMM) and amphotericin B (AmB) (Table 1). LEM3049 (MHOM/FR/95/LEM3049) originated from a French HIV co-infected patient; BH402/60 (MCAN/BR/2002/BH402/60) is a canine isolate from Brazil that was part of a large clinical trial with liposomal meglumine [23] while MHOM/BR/2007/WC (L3015) was obtained from a Brazilian HIV- patient [24]. The French clinical isolate LEM3323 (MHOM/FR/96/LEM3323) with a Sb-resistant background and its experimentally derived MIL-resistant (LEM3323/MIL) and PMM-resistant (LEM3323/PMM) lines [25] were specifically included to assess the discriminating capacities of the different cell types for drug resistant strains. Five recent *L*. *infantum* clinical isolates from the recent outbreak in Fuenlabrada (Madrid, Spain) were included in the study to further corroborate the selected assay system (MHOM/ES/2016/LLM-2301, LLM-2309, LLM-2323, LLM-2338, LLM-2346) [26]. Promastigotes were routinely cultured in M199 medium supplemented with HEPES, adenine, hemin, 6-biopterine, L-glutamine, 5% sodium bicarbonate and 10% heat-inactivated (56°C for 30 minutes) FBS (iFBS) [20]. Parasites were sub-cultured twice weekly and the passage number was kept as low as possible to avoid a decrease in virulence due to prolonged cultivation *in vitro* [27]. Five-day-old promastigote cultures were used for preconditioning, as described earlier [9].

**Table 1:** Overview of Leishmania infantum strains used. Origin and drug-susceptibility profile to the current antileishmania drugs (R: resistant; S: susceptible; [R]: resistant after experimental selection).

*L*.* infantum* strain	Code	Origin	Drug susceptibility profile			
			Sb^V^	Sb^III^	MIL	PMM
**ITMAP263**	MHOM/MA/67/ITMAP263	Laboratory reference strain	S	S	S	S
**LEM3049**	MHOM/FR/95/LEM3049	French field isolate from HIV^+^ patient	S	S	S	S
**L3015**	MHOM/BR/2007/WC	Brazilian field isolate from HIV^+^ patient	S	S	S	S
**LEM3323**	MHOM/FR/96/LEM3323	French field isolate from HIV^+^ patient	R	R	S	S
**LEM3323/MIL**		Experimentally selected MIL-resistant strain	R	R	[R]	S
**LEM3323/PMM**		Experimentally selected PMM-resistant strain	R	R	S	[R]

### Preparation of macrophages

A former comparison of mouse-derived primary peritoneal exudate cells (PECs) and bone marrow derived macrophages (BMMφ) already revealed some outcome differences [5, 28]. For in-depth evaluation of the impact of these findings on the susceptibility determination of clinical isolates, BALB/c mouse BMMφ and Swiss mouse PECs were seeded in 96-well plates, as previously described [28]. For the collection of PECs, Swiss mice were stimulated with a 2% starch suspension in PBS 24h prior to macrophage prelevation. PECs were collected via peritoneal lavage with 10mL RPMI-1640 macrophage medium, supplemented with 5% iFBS, 2% PS and 1% L-glutamine. Cells were counted in KOVA counting slides and seeded in 96-well plates at a final concentration of 30,000 cells/well. Bone marrow-derived macrophages (BMMφs) were obtained from the femur and tibia of female BALB/c mice by flushing the bone cavities with cold RPMI-1640 medium. The bone marrow was kept on ice during the isolation, then centrifuged for 20 minutes upon which the red blood cells were lysed with ammonium-chloride-potassium lysis buffer (VWR, Leuven, Belgium). The recovered cells were resuspended in RPMI-1640 medium supplemented with 1% non-essential amino acids, 1% P/S solution, 1% sodium pyruvate, 1% L-glutamine, 10% iFBS and 15% L929 supernatant containing macrophage colony stimulating factor (M-CSF), and incubated in Petri dishes at 37°C and 5% CO_2_ to obtain macrophage monolayers. After 7 days, the cells were detached from the dishes with dissociation buffer (1% 0.5M EDTA, 2% 1M HEPES in PBS), counted and seeded similarly to the PECs.

Human peripheral blood mononuclear cells (PBMCs) were isolated from the blood of healthy volunteers. A small volume of blood was collected in covered test tubes and allowed to clot 15–30 minutes at room temperature. The clot was separated by centrifugation at 2,000 x g for 10 minutes at 4°C upon which the serum was transferred into a clean tube and inactivated at 56°C for 30 minutes. For the isolation of PBMCs, blood was collected in heparin-coated tubes, layered onto Histopaque-1077 and centrifuged at 400×*g* for 30 minutes. After gradient separation, the cells were collected and washed three times with RPMI-1640 medium, counted in a KOVA counting chamber and suspended in RPMI-1640 supplemented with 2% P/S and 10% autologous human serum. Cultures were incubated at 37°C and 5% CO_2_ for 6 days to produce macrophage monolayers.

J774_A.1_ and RAW_264.7_ macrophage cell lines were purchased from ATCC and cultured in Dulbecco’s modified minimal essential medium (DMEM) supplemented with 10% iFBS. Cells were grown in T25 culture flasks at 37°C in presence of 5% CO_2_. Every 2 days, the cells were detached from the culture flask with a cell scraper and sub-cultured. The cells were counted and plated in 96-well plates at a density of 30,000 cells/well 24h before infection.

THP-1 human monocyte cell line was obtained from ATCC and cultured at 37°C and 5% CO_2_ in RPMI-1640 medium supplemented with 10% iFBS. The cells were sub-cultured every two days with particular attention not to exceed a cell density of 10^6^/mL. Before infection, 30,000 cells/well were plated in 96-well plates and simultaneously stimulated with 100 ng/mL PMA. The PMA stimulus was removed either after 48h by renewing the medium followed by incubation for another 5 days to allow complete macrophage differentiation [17], or after 72h immediately before infection.

### Receptivity for infection

To determine the macrophage’s receptivity for infection, the initial rate of phagocytosis was evaluated for each cell type. The infection index was determined microscopically at 2h, 4h and 6h post-infection upon Giemsa staining and was calculated as described previously [29]. In brief, the intracellular parasite burden was quantified microscopically in at least of 50 macrophages (both non-infected and infected), from which the infection index (average number of amastigotes/cell) was calculated.

### Parasite and cell replication

Intracellular amastigote replication was evaluated to assess the host cell supportive capacity for infection [25]. Briefly, infection indices of the different types of macrophages were determined microscopically during a 10-day course of experiment. Renewal of the culture medium was omitted to minimize interactions during culture and mainly because no significant decrease in cell viability was observed until up to 168 hpi (S4 Fig). To determine whether continuously dividing cell lines (J774_A.1_ and RAW_264.7_) could still be used to determine the IC_50_, their replication potential was cross-checked to the intracellular replication capacities of the parasites.

### Impact of intracellular parasite burden and drug exposure time on drug susceptibility

For each *L*. *infantum* isolate, the 50% inhibitory concentration (IC_50_) against the standard drugs was determined in each of the different cell types. Macrophages were infected at a ratio of 15:1 by pre-conditioned promastigotes of LEM3049 that was selected as drug-susceptible field strain. Macrophages were exposed 24h later to 2-fold drug dilutions of the reference drugs Sb^V^, Sb^III^, MIL, PMM and AmB. The IC_50_ value was determined microscopically 96 hours post-infection (hpi) upon methanol fixation and Giemsa-staining.

As several studies in literature used different drug exposure times for susceptibility determination [5, 30–32], the impact of exposure time on the IC_50_ was evaluated in parallel by determining the IC_50_ for each drug at 24 hour intervals post-treatment. As previous research also demonstrated that the infection index can impact on cell proliferation or survival [33] and hence may affect the IC_50_-value [14], a 2-fold dilution of the parasite inoculum combined with a 2-fold dilution of MIL (not done for the other drugs) was included for drug susceptibility determination at different infection indices.

### Capacity to discriminate drug resistance

Monitoring drug susceptibility in the field implies the need for a fast and reliable laboratory result. Given the cell-specific susceptibility [21], it is logical to assume that different macrophage types will not equally detect drug resistance against the current reference drugs (breakpoints as used in the present study are listed in Table 2). This was checked by inclusion of LEM3323 having a comparable background and drug susceptibility (except for Sb) to the drug-susceptible LEM3049 and the experimentally resistant selected LEM3323/MIL and LEM3323/PMM lines. The discriminating capacity of each cell type to detect drug resistance was evaluated by calculating the resistance index (RI) for each drug (IC_50_ resistant strain/IC_50_ susceptible strain). The higher the RI, the higher the discriminatory capacity of the cell to identify resistance in the field.

**Table 2:** ‘Breakpoint’ estimates^#^ for categorizing drug-susceptibility and drug resistance for clinical isolates. [taken from ref 20].

Drug	Promastigotes (axenic)	Amastigotes (primary mouse macrophages)	Susceptibility limits (estimates)		Cytotoxicity (MRC5)
	approx IC_50_		Sensitive	Resistant	CC_50_
**Sb**^**V**^	>77	10–15	<20	>70	>64
**Sb**^**III**^	40–50	5–6	<15	>70	>64
**MIL**	2–5	3–6	<10	>25	32
**PMM**	15–25	40–50	<60	>150	>500
**AmB**	0.1–0.3	0.01–0.03	<0.5	(>2)*	>8

^#^ based on results obtained with sensitive reference strains (*L*. *donovani*: MHM/ET/67/L82 and *L*. *infantum* MHOM/MA/67/ITMAP263)

* AmB-resistant isolates from treated patients are yet not available

CC_50_: cytotoxic concentration 50%

### Comparison of different approaches for the drug susceptibility evaluation of clinical isolates

Based on the obtained results and recommendations on drug exposure time (120h), optimal infection index (≥10:1) and problems with rapid cellular replication, the drug susceptibility of five recent European *L*. *infantum* clinical isolates (MHOM/ES/2016/LLM-2301, LLM-2309, LLM-2323, LLM-2338, LLM-2346) was determined and compared upon infection of THP-1 cells, BMMφ and PECs.

### Statistical analysis

All statistical analyses were performed using Graphpad Prism version 6.00 software. Statistical differences between the infection ratio of different *Leishmania* strains at different time points were determined using 2-way ANOVA with Bonferroni post-hoc comparisons. Tests were considered statistically significant if *p* <0.05.

## Results

### Receptivity for infection

**Fig 2:**
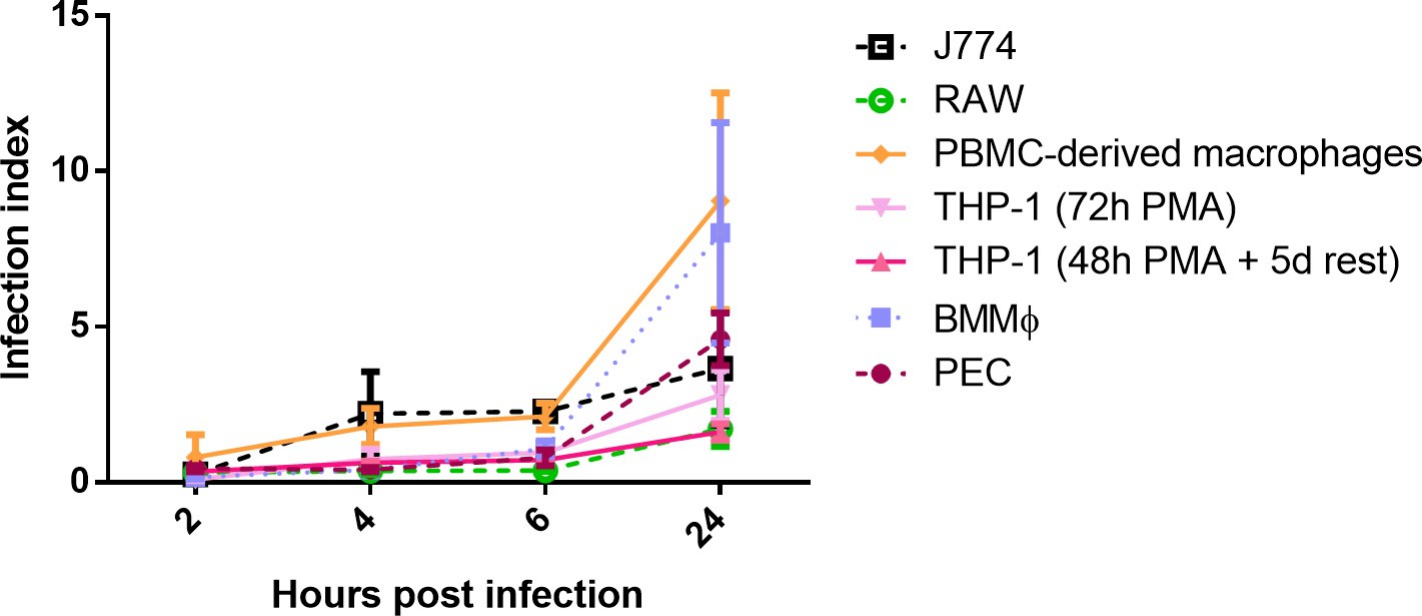
Promastigote phagocytosis by the different cell types upon infection with the laboratory strain ITMAP263. Results are expressed as the average infection indices at 2h, 4h, 6h and 24hpi ± standard error of the mean (SEM) and are the result of two independent experiments run in duplicate.

For each cell type, the receptivity to infection was evaluated by comparing initial phagocytosis rates (S1 Fig). For each *Leishmania* strain, a same trend of fast uptake by primary cells could be observed: PBMC-derived macrophages demonstrated the quickest uptake, followed by the PECs and BMMφ (Fig 2). The phagocytosis levels in THP-1 cells were slightly higher compared to RAW and J774 cells.

### Parasite burden and cell replication

**Fig 3:**
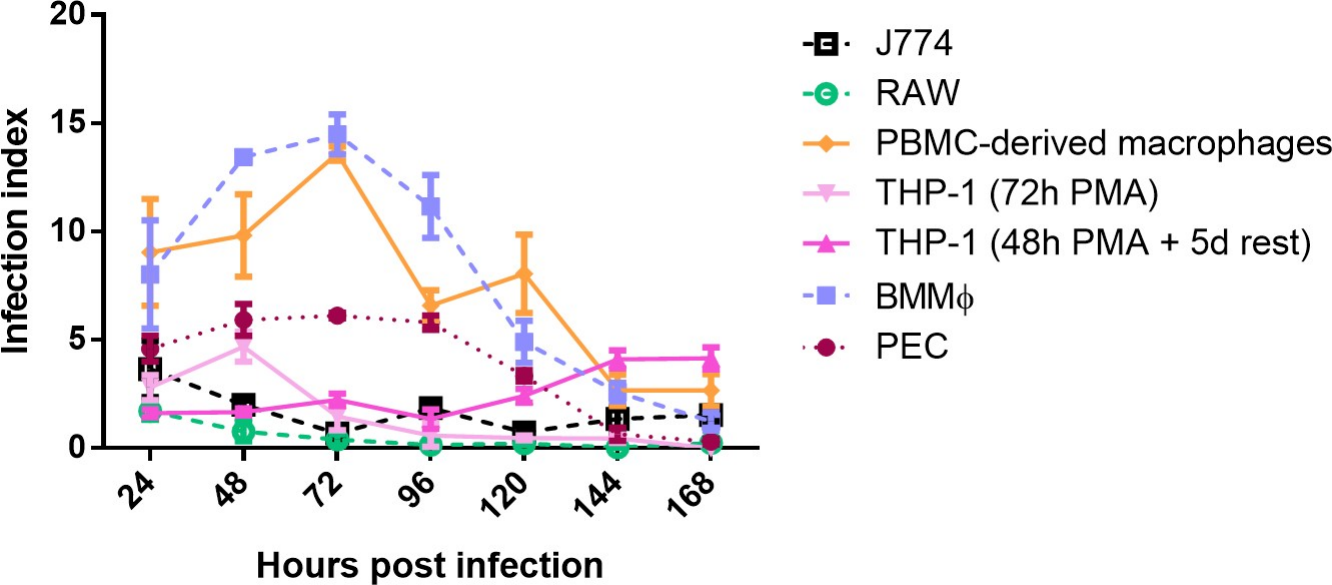
Intracellular amastigote multiplication of the laboratory reference strain ITMAP263. The number of amastigotes/macrophages (infection index) was determined until 168hpi. The average infection index ± SEM is the result of two independent experiments run in duplicate.

The intracellular proliferation of the *Leishmania* isolates was evaluated in the selected panel of host cells (S2 Fig). As the results between strains were fairly comparable, only the results of the laboratory reference strain ITMAP263 are presented. The higher amastigote survival and replication in BMMφ had already been demonstrated in relation to other primary cell types [28]. Here, PBMC-derived macrophages reach comparable initial infection indices, hereby identifying BMMφ and PBMC-derived macrophages to provide the better support for amastigote survival and replication, followed by PEC. For LEM3049 and BH402/6, intracellular multiplication was better in PEC, demonstrating some strain-dependent effect. Infection indices are systematically lower in the cell lines compared to PEC (Fig 3).

**Fig 4:**
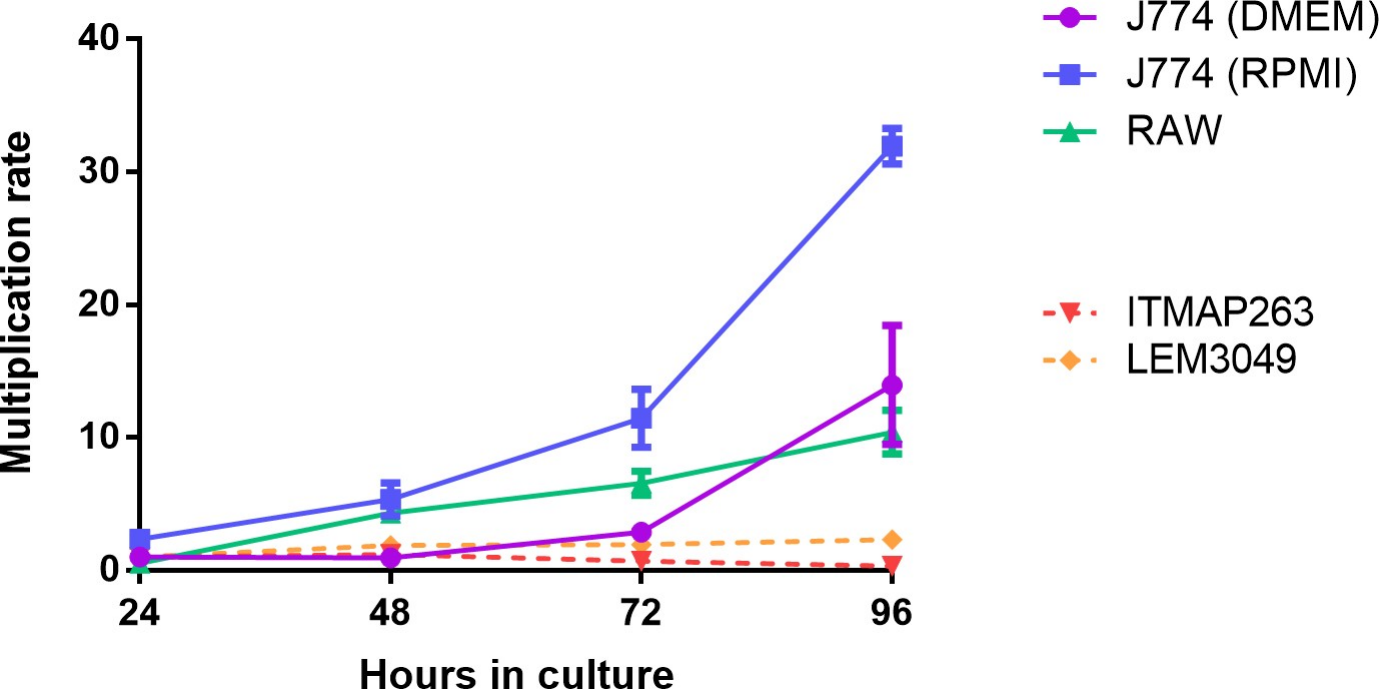
Multiplication rate of J774 and RAW cells in relation to the intracellular growth of ITMAP-263 (not showing significant intracellular multiplication in PECs) and LEM3049 (showing rapid intracellular replication in PECs). Although both Leishmania strains demonstrate intracellular replication, parasitized cells become outcompeted because of the more rapid expansion of non-infected host cells, resulting in low infection ratios. Multiplication rates are expressed as the average ± SEM of two independent experiments run in duplicate.

Evaluating the intracellular growth/multiplication rate of parasites in the continuously dividing cell lines proved to be challenging. The rapidly dividing cells tend to outgrow the parasitized cell population within 48h, even for LEM3049 known to have a high multiplication capacity in non-dividing primary cells. The observation that various studies use RPMI-1640 medium to cultivate J774 cells in contrast to the DMEM medium as recommended by ATCC prompted us to compare the multiplication rate of J774 in both media. The J774 multiplication rate was highest in RPMI (Fig 4).

### Drug susceptibility determination and capacity to discriminate drug resistance

The drug susceptibility of LEM3049 in the various macrophage cell types is shown in Table 3. Variation in drug susceptibility is noted between the different cell types with primary cells showing higher RI values, meaning they may allow easier detection of resistance for the selected panel of reference drugs. The cell types with RI values approximating 1 are less suited for detection of drug resistance. Similar to the IC_50_-values, the RI is drug-dependent and no particular added benefit can be noted for one particular cell type. In THP-1 cells, the PMA stimulation period and the 5-day period between stimulation and infection did not affect drug susceptibility, except for Sb^III^ where the efficacy significantly increased (p = 0.0006) with longer PMA-stimulation before infection.

**Table 3:** Drug-susceptibility and resistance indices (RI) of the drug-susceptible LEM3049 isolate for the antileishmanial reference drugs in the different macrophage cell types. The 50% inhibitory concentration (IC_50_) of LEM3049 and the RI against pentavalent and trivalent antimonials (Sb^V^ and Sb^III^), miltefosine (MIL), paromomycin (PMM) and amphotericin B (AmB) were determined in primary peritoneal Swiss mouse macrophages (PECs), BALB/c bone-marrow derived macrophages (BMMφ), human blood mononuclear cell-(PBMC) derived macrophages and the commercially available THP-1 and J774 cell lines (*not done for RAW cells*). Values are expressed as the average IC_50_ ± SEM and are the result of two independent tests run in duplicate.

Host cell type	Antileishmanial reference drug: IC_50_ ± SEM																			
	Sb^V^ (eq.)			*RI*	Sb^III^ (eq.)			*RI*	MIL (μM)			*RI*	PMM (μM)			*RI*	AmB (μM)			*RI*
**Primary cells**																				
PEC	52.4	±	24.6	***1*.*5***	6.3	±	0.4	***14***	0.5	±	0	***80***	99.3	±	10.3	***1*.*3***	0.05	±	0.01	***ND***
BMMφ	>77			***1***	11.1	±	1.5	***7*.*9***	0.5	±	0.1	***70*.*3***	25.3	±	7	***6***	0.05	±	0.02	***ND***
Human PBMC-derived macrophages	>77			***2***	7.9	±	0.1	***11*.*1***	1.3	±	0.2	***27*.*4***	113	±	9.6	***4*.*4***	0.07	±	0.01	***ND***
**Cell lines**																				
THP-1 (48h PMA + 5d rest)	>77			***1***	30.2	±	5.8	***2*.*1***	1.8	±	0.1	***11*.*2***	37.2	±	13.9	***6*.*1***	0.16	±	0.02	***ND***
THP-1 (72h PMA)	>77			***2***	9.5	±	0.7	***4*.*6***	1.3	±	0.4	***15*.*4***	39.1	±	4	***5*.*3***	0.12	±	0	***ND***
J774	5.5	±	0	***ND***	>22			***ND***	2.2	±	0.7	***ND***	15.4	±	4.3	***ND***	0.01	±	0	***ND***

### The impact of drug exposure time and intracellular parasite burden on drug susceptibility

**Fig 5:**
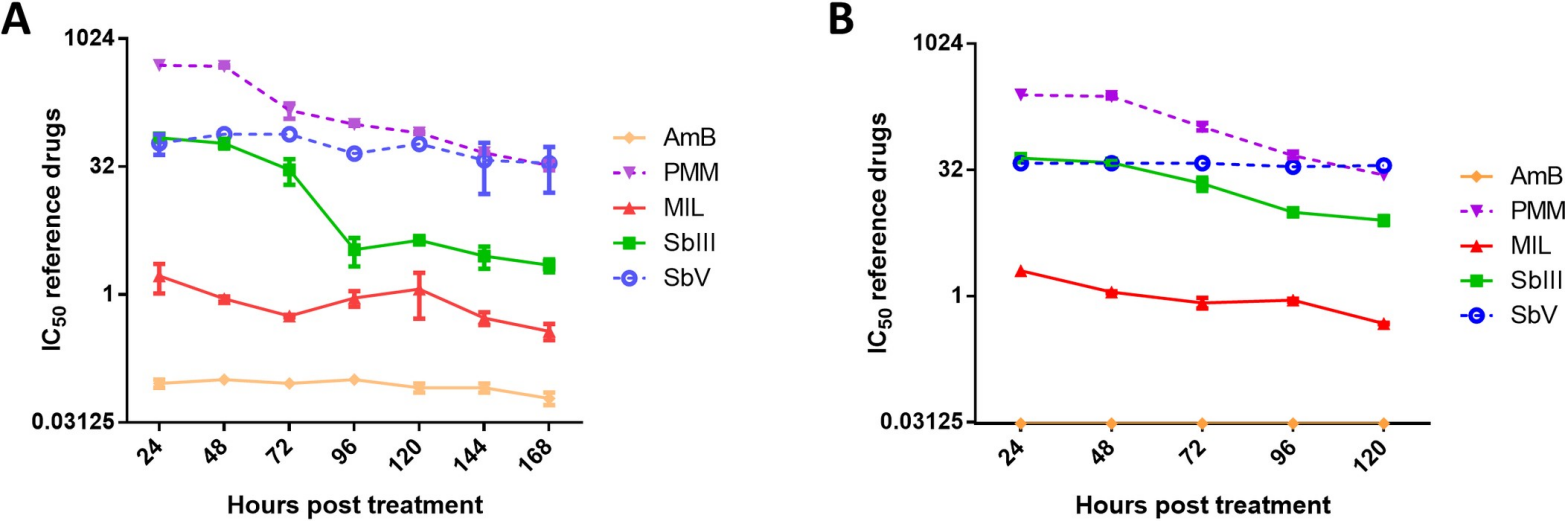
Effect of drug exposure time on parasite drug susceptibility. The 50% inhibitory concentration (IC_50_) of the clinical isolate LEM3049 for the reference drugs in Swiss mouse peritoneal macrophages (A) or in BALB/c bone marrow-derived macrophages (B) in function of treatment duration. The cell viability at the IC_50_ levels at 24h and at 168h remained >95%. Drug susceptibility is expressed as average IC_50_ values (μM for MIL, PMM and AmB; eq. for Sb^V^ and Sb^III^) ± SEM for two independent tests run in duplicate. (Sb^V^: pentavalent antimony, Sb^III^: trivalent antimony, MIL: miltefosine, PMM: paromomycin, AmB: amphotericin B).

**Fig 6:**
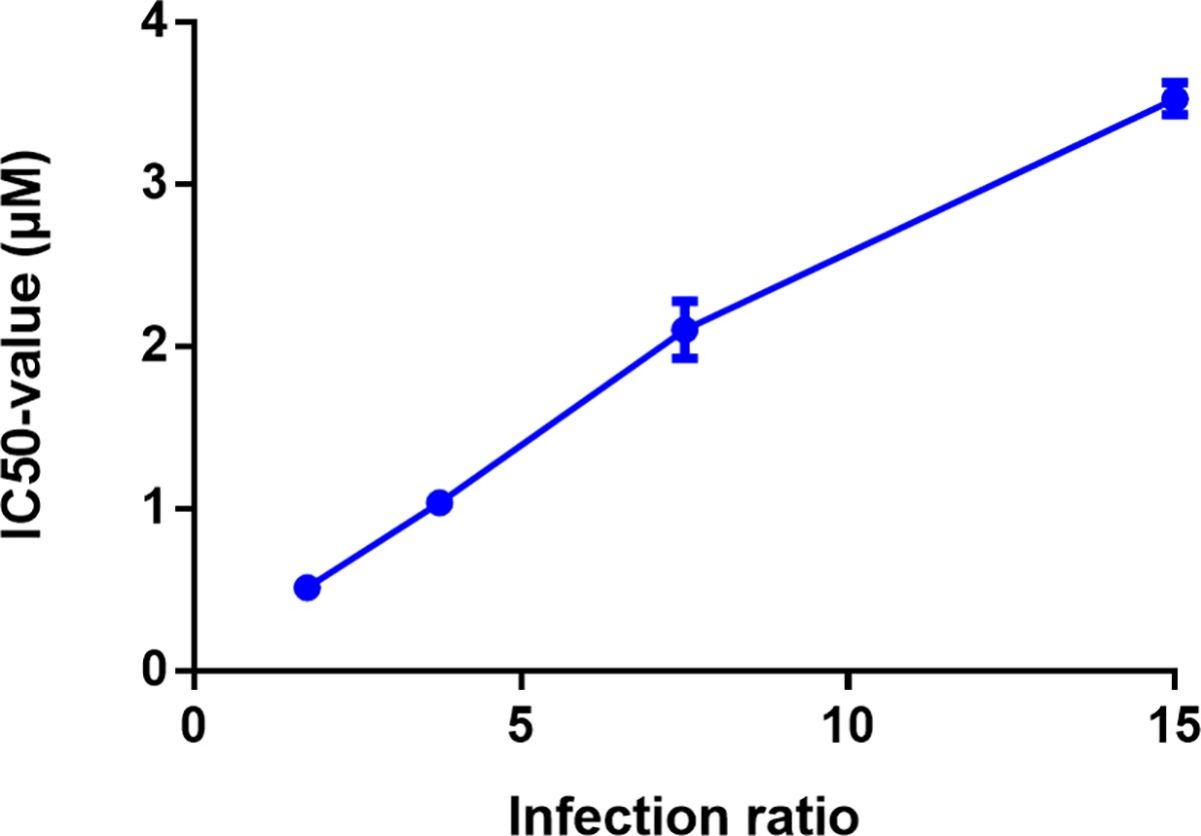
Effect of the infection ratio on the miltefosine IC_50_-value of the clinical isolate LEM3049 in Swiss mouse peritoneal macrophages (PECs) at 120hpt. Drug-susceptibility values are expressed as average IC_50_ value (*μ*M) ± SEM and are the result of two independent tests run in duplicate.

To evaluate the drug exposure time required to reach a stable IC_50_, the shift in drug IC_50_ for the various reference drugs over time is shown in Fig 5A. For MIL, Sb^V^ and AmB, no significant differences were observed between the IC_50_ values measured at different time points post-treatment. For Sb^III^ and PMM, a significant decrease was observed with stable IC_50_ values respectively reached at 120 and 144 hours post-treatment (hpt). A same trend was also observed in BMMφ (Fig 5B). Noteworthy is that the infection ratio can significantly impact on drug susceptibility measured at 120 hpt. For example for MIL, the higher infection burdens obtained after infection with higher infection ratios were associated with lower observed drug susceptibility (Fig 6). Although not investigated here, it is acceptable to assume that the same will apply to the other antileishmanial drugs.

### Comparison of different approaches for the drug susceptibility evaluation of clinical isolates

The intracellular drug susceptibility was determined for five recent clinical isolates using the preferred methodology, *i*.*e*. primary macrophages or THP-1 cells, infection with promastigotes at 10:1 multiplicity of infection and 120 h of drug exposure (Table 4). Although some variability in drug susceptibility can be noted between the different host cells, the overall susceptibility profile was comparable for the three cell types. Variation in IC_50_-values was observed only for PMM between the different cells which might obscure the identification of PMM-resistant isolates (resistance cut-off 150 μM) [22]. The higher IC_50_-values against Sb^V^ demonstrated in PEC may also complicate the identification of Sb-resistant strains.

**Table 4:** *In vitro* drug susceptibility of a panel of recent clinical *L*. *infantum* isolates against antileishmanial reference drugs in THP-1, bone-marrow derived macrophages (BMMφ) and mouse peritoneal exudative cells (PEC). Intracellular amastigote drug susceptibility against miltefosine (MIL), paromomycin (PMM), pentavalent antimonials (Sb^V^), trivalent antimonials (Sb^III^) and amphotericin B (AmB). The presented IC_50_-values are the result of three independent tests run in duplicate.

Strain	*In vitro* susceptibility of intracellular amastigotes (IC_50_ ± SEM)														
	MIL (μM)			PMM (μM)			Sb^V^ (μg/ml eq)			Sb^III^ (μg/ml eq)			AmB (μM)		
	*mean*	*±*	*SEM*	*mean*	*±*	*SEM*	*mean*	*±*	*SEM*	*mean*	*±*	*SEM*	*mean*	*±*	*SEM*
**THP-1**															
LLM-2301	0.9	±	0	136.2	±	21.6	33.6	±	20.8	14.4	±	4.6	0.12	±	0.02
LLM-2309	0.1	±	0	29	±	8.4	50.8	±	12.5	7.6	±	2.6	0.04	±	0.01
LLM-2323	0.4	±	0.1	55	±	8	49.8	±	14.9	10.6	±	5	0.06	±	0.02
LLM-2338	0.4	±	0.1	324.6	±	104.2	43	±	20.1	7.8	±	3	0.10	±	0.01
LLM-2346	0.5	±	0.2	87.8	±	26.8	18.8	±	3	6.8	±	0.1	0.10	±	0.01
**BMM**φ															
LLM-2301	0.3	±	0.1	157.7	±	45.5	54.9	±	10.4	12.3	±	1.8	0.05	±	0.01
LLM-2309	ND			ND			ND			ND			ND		
LLM-2323	0.4	±	0.1	169.1	±	37.1	58.9	±	11.8	3.4	±	0.3	0.02	±	0.01
LLM-2338	0.3	±	0	276	±	42.1	67.7	±	9.3	9.2	±	3.2	0.05	±	0.02
LLM-2346	3.7	±	1.7	365.7	±	97.2	65.7	±	11.3	6.4	±	0.8	0.09	±	0.01
**PEC**															
LLM-2301	0.9	±	0.4	354.9	±	60	58	±	15.5	23.5	±	9.7	0.09	±	0.04
LLM-2309	0.1	±	0	25.7	±	5.7	58.8	±	4.4	8.4	±	1.4	0.04	±	0.01
LLM-2323	0.5	±	0.2	274.3	±	75.5	52	±	12.4	24.4	±	14.3	0.05	±	0.02
LLM-2338	0.6	±	0.2	278.1	±	70.3	52	±	14.4	7.2	±	2.2	0.05	±	0.01
LLM-2346	3	±	0.5	218.1	±	58.2	>77			8.7	±	2.1	0.10	±	0.01

## Discussion

Inter-laboratory comparison of drug susceptibility data proves to be very difficult given the large number of laboratory procedures, and although most researchers now agree on the use of amastigote cell-based assays [5], the availability of several host cells, both primary and cell lines, further complicates the progress towards harmonization. Despite earlier recommendations [19, 20], it remains essential to corroborate previous findings and to identify the best suited host cell for rapid and reproducible detection of drug resistance in view of the growing numbers of treatment failures against the current antileishmanial drugs [34–36]. The present laboratory study specifically aimed at comparing the advantages and disadvantages associated with a variety of frequently used primary macrophages and commercially available cell lines next to some important procedure-related differences in an attempt to propose a pragmatic drug susceptibility assay for (clinical) visceral *Leishmania* isolates. Although it can be assumed that some of the present assay recommendations for VL may also be translatable to the different cutaneous *Leishmania* species/strains, such cross-check should still be investigated in detail as it fell beyond de scope of the present study.

A first outline for an *in vitro* assay to determine drug activity against the intracellular *L*. *donovani* amastigotes was already published in 1984 [14] demonstrating that the determination of a reproducible drug susceptibililty value is hampered by the macrophage infection ratio (average number of amastigotes/macrophage) which not only influences intracellular amastigote proliferation but also has a direct impact on the measured activity level of the drug. It was also the first description on the use of primary macrophages from the perspective that these may more closely mimic the *in vivo* environment of the pathogen in a more reproducible way. Within that context, the implementation of several *ex vivo* models in drug screening applications has been investigated [37–39]. Although peritoneal macrophages loaded with amastigotes do not fully represent the lesion cell, they are still considered the easiest, cheapest, quickest primary model to evaluate antileishmanial compounds. On the other hand, using primary cells is associated with ethical constraints, cells do not retain their *in vivo* functionality and morphology, cell storage is impossible and one cannot rule out the possible contamination by other cells. For example, when harvesting human monocyte-derived macrophages, only 10% of the peripheral blood monocytes will attach and transform into functional macrophages [17]. This factor combined with the difficult logistics of using human-derived primary cells do limit their use in large-scale evaluation campaigns. Although cell lines are physiologically less relevant to mimic the *in vivo* situation, they are a relatively cheap alternative to the use of primary cells with much less ethical restrictions. In addition, cell lines have a homogeneous background, stable phenotype and above all a long life-span allowing a high degree of reproducibility. However, repeated passage *in vitro* may alter their surface-structure, THP-1 cells require a lot of manipulation, immune activation is cell-type dependent [40] and their high replication potential complicates maintenance of a stable parasite infection over time (Fig 4), also leading to practical difficulties such as staining issues that obscure microscopic read-out.

Regardless of the cell type associated advantages and disadvantages (Table 5), identifying the better suited host cell for easy and reproducible detection of drug resistance in the field becomes a more urgent necessity given the growing incidence of therapy failure and drug resistance. Our starting approach was to comparatively evaluate drug susceptibility with the susceptible field isolate LEM3049 in each cell type and to determine the RI-values using experimentally generated resistant strains. Marginal host cell dependent variations in drug susceptibility could be noted, which is in agreement with another study comparing different cell types [21]. The observation of higher RI-values in the primary cells could imply higher sensitivity and specificity to detect resistance.

**Table 5:** Summary of advantages and disadvantages of the selected panel of host cells for *Leishmania* susceptibility testing.

Cell type	Primary cells			Cell lines		
	PEC	BMMφ	PBMC	THP-1	J774	RAW
	*Peritoneal macrophages*	*Myeloid progenitor cell derived*	*Monocyte* *derived macrophages*	*human* *monocyte* *derived*	*BALB/c* *monocyte* *derived*	*BALB/c macrophage derived*
**Source**	Mouse	Mouse	Human blood	Commercial	Commercial	Commercial
**Cell stimulation**	starch or thioglycollate	L929 supernatant GM-CSF	NA	PMA, VitD_3_ or RA	NA	NA
**Approximate yield**	10^7^ cells/mouse	10^7^−10^8^ cells/mouse	10^5^−10^6^/mL (10% monocytes)	NA	NA	NA
**Ethical concerns**	+++	+++	++	-	-	-
**Biological relevance**	++	++	+++	-	-	-
**Receptivity for infection**	+++	+++	+	+	++	++
**Intracellular multiplication**	+	++	++	+	+/-	+/-
**Reproducibility**	+++	+++	+/-	+/-	++	++
**Cost**	++	++	+/-	+/-	+/-	+/-
**Consumables needed**	+	++	+++	+	+	+
**Technical complexity**	+	+++	++	+/-	+/-	+/-

The study results also suggest that an exact IC_50_-value will to some extent depend on the nature of the host cell and the strain phenotype. Another question is whether drug susceptibility data collected on mice-derived host cells are fully translatable to human. The use of mouse-derived cells may indeed fail to demonstrate subtle variations in drug susceptibility, but their application in drug susceptibility campaigns has proven their value in identifying ‘clear resistant’ phenotypes of the parasite. In contrast to a previous study using spleen-derived amastigotes for infection [21], both BMMφ and PBMC-derived macrophages sustained intracellular replication for the whole panel of strains upon infection with metacyclic promastigotes. Earlier work with PECs already demonstrated different intracellular growth kinetics depending on the infecting parasite stage [25, 29].

Most laboratories prefer *ex vivo* amastigotes to infect cells as they provide higher and more consistent infection ratios in contrast to metacyclic promastigotes. Acceptable infections are generally obtained using an amastigote/macrophage ratio between 5 and 10 [14], but the amastigote quantification method as described by Stauber [14] may result in quite variable infection ratios *in vitro*. Particularly for clinical isolates, even upon exact microscopic counting of viable stages before infection, large fluctuations in infection ratios may occur due to the unpredictable intrinsic variation in parasite virulence and intracellular multiplication potential. Generating infective metacyclic promastigotes of clinical isolates remains a challenge since strain-specific differences will lead into different growth rates and ensuing metacyclogenesis rates. Promastigote preconditioning [9] has been proposed as an option to alleviate these variabilities to achieve more adequate infection ratios of clinical isolates. The promastigote medium is manually acidified 24h before infection enforcing metacyclogenesis in a more controlled way and thereby allowing simultaneous (comparative) infection of strains with different growth characteristics. The impact of preconditioning on the different strains was not part of the present study. Although HOMEM or RPMI-1640 are frequently used for promastigote cultivation, M199 medium was selected to cultivate the different clinical isolates as a cheaper alternative supporting the growth of most clinical isolates [22]. Attention should be given to keep the lag time between primary isolation and drug susceptibility assays to a minimum to avoid loss of virulence and other phenotypic alterations caused by long-term *in vitro* cultivation. Logically, the more rapid assay result will also benefit the proper therapeutic follow-up of the patient.

Comparison of the intracellular growth in different cell lines already demonstrated that LEM3049 is able to divide in PECs, while BH402/60 and ITMAP263 did not [25]. Quite similar trends were observed for the different cell types tested here: PBMC-derived macrophages and BMMφ show a rapid parasite internalization together with intracellular parasite multiplication, while phagocytosis and proliferation in PECs remains highly strain-specific, which again illustrates the complexity of the interaction between host cell and parasite.

It has already been demonstrated that the potency of SSG (Sb^V^) decreased with increasing parasite burdens [14], explaining the higher IC_50_ observed for Sb^V^ in PECs. Here, a same trend can be observed for MIL. Similarly to the infection ratio, the percentage infected cells can also confound correct drug susceptibility determination as low infection levels will result in unreliable IC_50_ read-outs. A general rule should be that at least over 80% of the macrophages should be infected in the positive control before even considering defining susceptibility values. When taking this rule into account, the susceptibility read-out of the cell lines becomes severely compromised. While the infection ratio stays lower in THP-1 cells, the extensive cell proliferation of RAW and J774 causes a rapid decrease of the infection ratio (Fig 3). The intracellular replication of the four isolates in the present study was most evident in human PBMC-derived cells and BALB/c BMMφ (S2 Fig) with the highest infection ratio being reached at 24 hpi. While the exact causes of cell-dependent drug susceptibility still remain elusive, the cell differentiation rate may be one factor that can influence promastigote phagocytosis and subsequent promastigote-to-amastigote transformation. The time-dependent efficacy of the antileishmanial reference drugs advocates for treatment periods of at least 96 h in primary cells. Combining the need for prolonged drug exposure with the observed low infection rates obtained in the cell lines makes them less recommendable for drug susceptibility determination.

A final step in the procedure is the read-out, which for clinical isolates will generally remain dependent of light microscopy. Defining *in vitro* efficacy can in principle be done in two ways: (i) counting and calculating the percentage reduction of the average number of amastigotes per macrophage, or (ii) determining the reduction in the average percentage of infected cells. While the latter is simpler and less time-consuming, the former is more precise and can be particularly handy in case of near-toxicity [14].

In summary (Table 6), our results recommend the use of primary cells in drug susceptibility evaluation whenever possible. Although intracellular replication is more pronounced in PBMC-derived macrophages and BMMφ, their limited availability and technically challenging purification and transformation procedures will severely limit their application in large-scale settings. Given the narrow variation in IC_50_ values and RI-values between PBMCs-derived cells, BMMφ and PECs, we propose PECs as a pragmatic option. Intraperitoneal stimulation of outbred Swiss or CD-1 mice strongly enhances the macrophage yield and parasite phagocytosis [14].

**Table 6:** Summary of the proposed strategy for drug susceptibility testing of clinical isolates and/or laboratory-adapted strains.

*Leishmania* strain	Field isolate	Laboratory strain Lab-adapted field isolate
**Host cell types**	Mouse PECs (after starch stimulation) THP-1 cells (after PMA stimulation)	PEC (Swiss ~ BALB/c) BMMφ BALB/c: higher intracellular multiplication PBMC-derived macrophages (GM-CSF stimulation)
**Parasite stage**	Metacyclic promastigotes (preconditioning optional)	*Ex vivo* (spleen-derived) amastigotes
Multiplicity of infection^*^	10–15 promastigotes/cell	5–10 amastigotes/cell
**Start drug exposure**	24h after infection	2h after infection
**Duration drug exposure**	120h	120h

* dependent on the intrinsic infectivity of the strain

If practical or ethical limitations would impose the application of a cell line, THP-1 cells are the best option (Table 5). Despite their relatively low susceptibility for infection and the need to apply exogenous stimuli for monocyte-to-macrophage transformation, they provide an easy accessible and reproducible alternative to primary cells. However, a standard stimulation procedure still needs to be decided to avoid inter-laboratory bias and to ensure adequate macrophage transformation before infection. Although J774 and RAW cells are more robust, require less manipulation and are less sensitive to all sorts of stress during cultivation, their continuous replication severely compromises evaluation of slower-acting drugs.

While the focus of this laboratory study was the comparative evaluation of cell-based drug susceptibility assays, evaluation of field isolates in disease-endemic countries may prove to be problematic because of the reliance on dedicated laboratory infrastructure and advanced technical expertise. On the other hand, the conclusions of this study will be practically useful for investigators (in academia, hospitals, central field labs) who have access to the appropriate lab infrastructure and logistics.

## Supporting information

S1 Text Comparison of cell-based methodological approaches to determine drug susceptibility of visceral *Leishmania* isolates. (DOCX)

S1 Fig Intracellular multiplication rate of four *Leishmania* strains in Swiss peritoneal mouse macrophages. Multiplication rates are determined by microscopically determining their average infection index every 24h after infection in relation to the initial infection index at 24h post infection. Results are expressed as average multiplication rate of the standard error of the mean (SEM) of two independent experiments run in duplicate.
(TIF)

S2 Fig Cell infection of four *Leishmania* strains in the selected panel of host cells. Initial phagocytosis of 4 different *L*. *infantum* lines (A/ ITMAP263 laboratory reference strain and clinical isolates B/ LEM3049, C/ BH402/60 and L3015) is represented by microscopically determining their average infection index at 2h, 4h, 6h and 24hpi ± the standard error of the mean (SEM) of two independent experiments run in duplicate.
(TIF)

S3 Fig Amastigote multiplication rates of four *Leishmania* strains in a selected panel of host cells. Intracellular amastigote proliferation of 4 different *L*. *infantum* strains (A/ ITMAP263 laboratory reference strain and clinical isolates B/ LEM3049, C/ BH402/60 and L3015) is measured by microscopically determining the average infection index ± SEM in the different cell types every 24h up to 168 hours post-infection (hpi) of two independent experiments run in duplicate.
(TIF)

S4 Fig Cell viability of primary mouse peritoneal macrophages over time. **C**ell viability was determined by microscopic assessment of cell death upon trypan-blue staining. The average cell viability ± SEM is the result of two independent repeats run in duplicate.
(TIF)
