# Supplementary material for: In-depth comparison of cell-based methodological approaches to determine drug susceptibility of visceral Leishmania isolates
Source: PLoS Negl Trop Dis. 2019 Dec 2;13(12):e0007885. doi: 10.1371/journal.pntd.0007885 (PMC6907865; doi:10.1371/journal.pntd.0007885)
Supplement: S1 Text — (DOCX) [file pntd.0007885.s001.docx]

**S1 SUPPORTING INFORMATION**

Four different *Leishmania* strains (one laboratory reference strain and three clinical isolates), each with their own strain-specific growth characteristics (S1 Fig.), have been compared for their behavior in a panel of different host cells selected based on their established use in the field. To evaluate the host cell’s susceptibility to infection for each strain, parasite phagocytosis rates were compared between the different cell types. Initial parasite uptake was monitored microscopically by determination of the infection indices at 2h, 4h, 6h and 24hpi (S2 Fig.). Although some minor strain-specific effects can be observed, especially in PECs, the general conclusion is that phagocytosis rates are highest in BMMφ’s and PBMC-derived macrophages. To assess the host cell’s supportive role the infection, intracellular amastigote replication was evaluated during the course of the experiment (up to 168hpi) by microscopic evaluation of the infection burdens every 24h (S3 Fig.). Here, the former mentioned strain-specific nature of the PECs is even more pronounced. While for ITMAP263 and L3015, both reasonably avirulent strains *in vitro*, the highest parasite proliferation was noted in PBMC-derived macrophages and BMMφ, the PECs appear to be the best supporting host cell for both LEM3049 and BH402/60. Renewal of the culture medium was omitted to minimize interactions during culture and mainly because no significant decrease in cell viability was observed until up to 168 hpi (S4 Fig.).
