# Supplementary figures and images for: In-depth comparison of cell-based methodological approaches to determine drug susceptibility of visceral Leishmania isolates
Source: PLoS Negl Trop Dis. 2019 Dec 2;13(12):e0007885. doi: 10.1371/journal.pntd.0007885 (PMC6907865; doi:10.1371/journal.pntd.0007885)

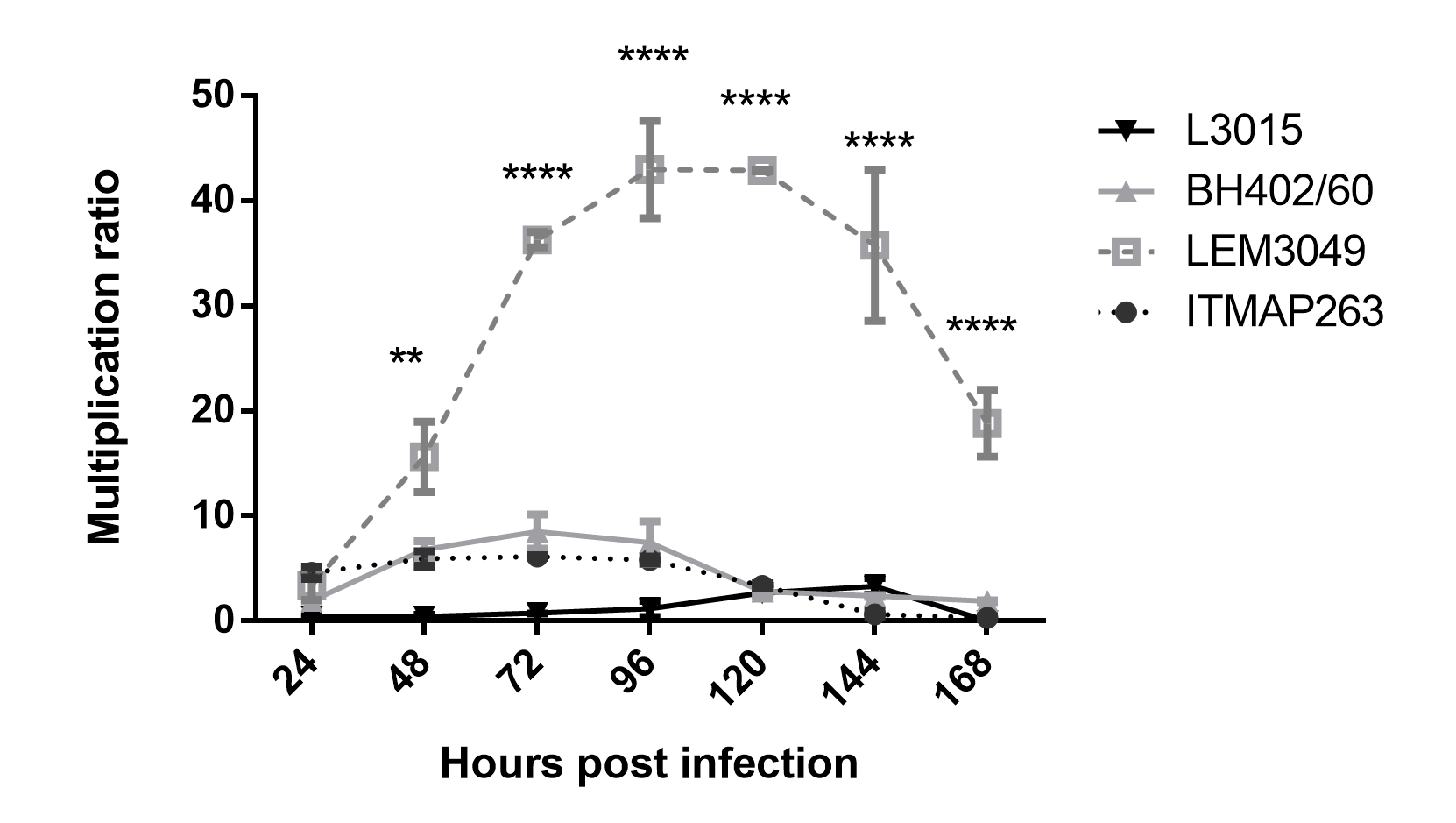

Supplement: S1 Fig — Multiplication rates are determined by microscopically determining their average infection index every 24h after infection in relation to the initial infection index at 24h post infection. Results are expressed as average multiplication rate of the standard error of the mean (SEM) of two independent experiments run in duplicate. (TIF) [file pntd.0007885.s002.tif]

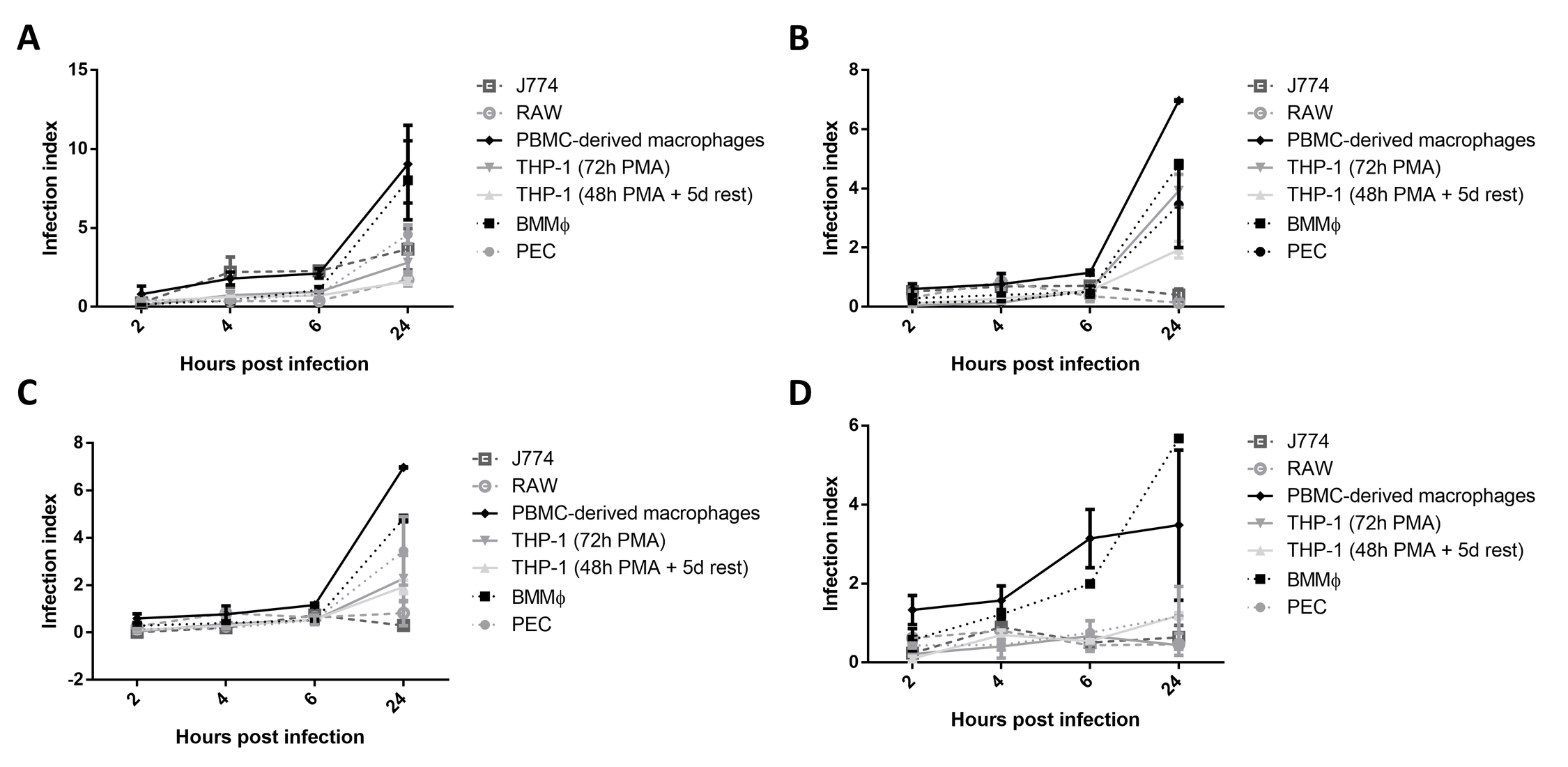

Supplement: S2 Fig — Initial phagocytosis of 4 different L. infantum lines (A/ ITMAP263 laboratory reference strain and clinical isolates B/ LEM3049, C/ BH402/60 and L3015) is represented by microscopically determining their average infection index at 2h, 4h, 6h and 24hpi ± the standard error of the mean (SEM) of two independent experiments run in duplicate. (TIF) [file pntd.0007885.s003.tif]

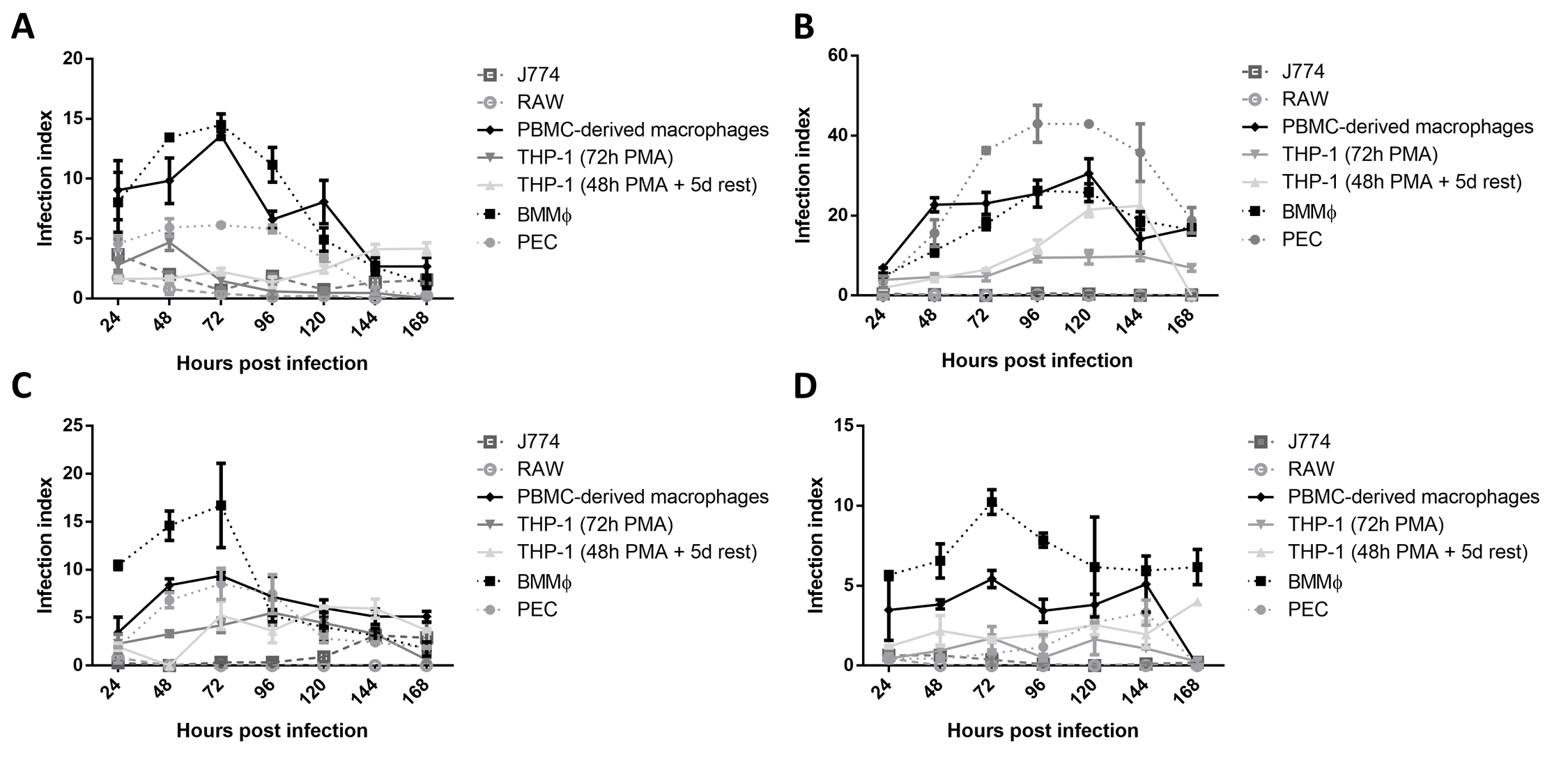

Supplement: S3 Fig — Intracellular amastigote proliferation of 4 different L. infantum strains (A/ ITMAP263 laboratory reference strain and clinical isolates B/ LEM3049, C/ BH402/60 and L3015) is measured by microscopically determining the average infection index ± SEM in the different cell types every 24h up to 168 hours post-infection (hpi) of two independent experiments run in duplicate. (TIF) [file pntd.0007885.s004.tif]

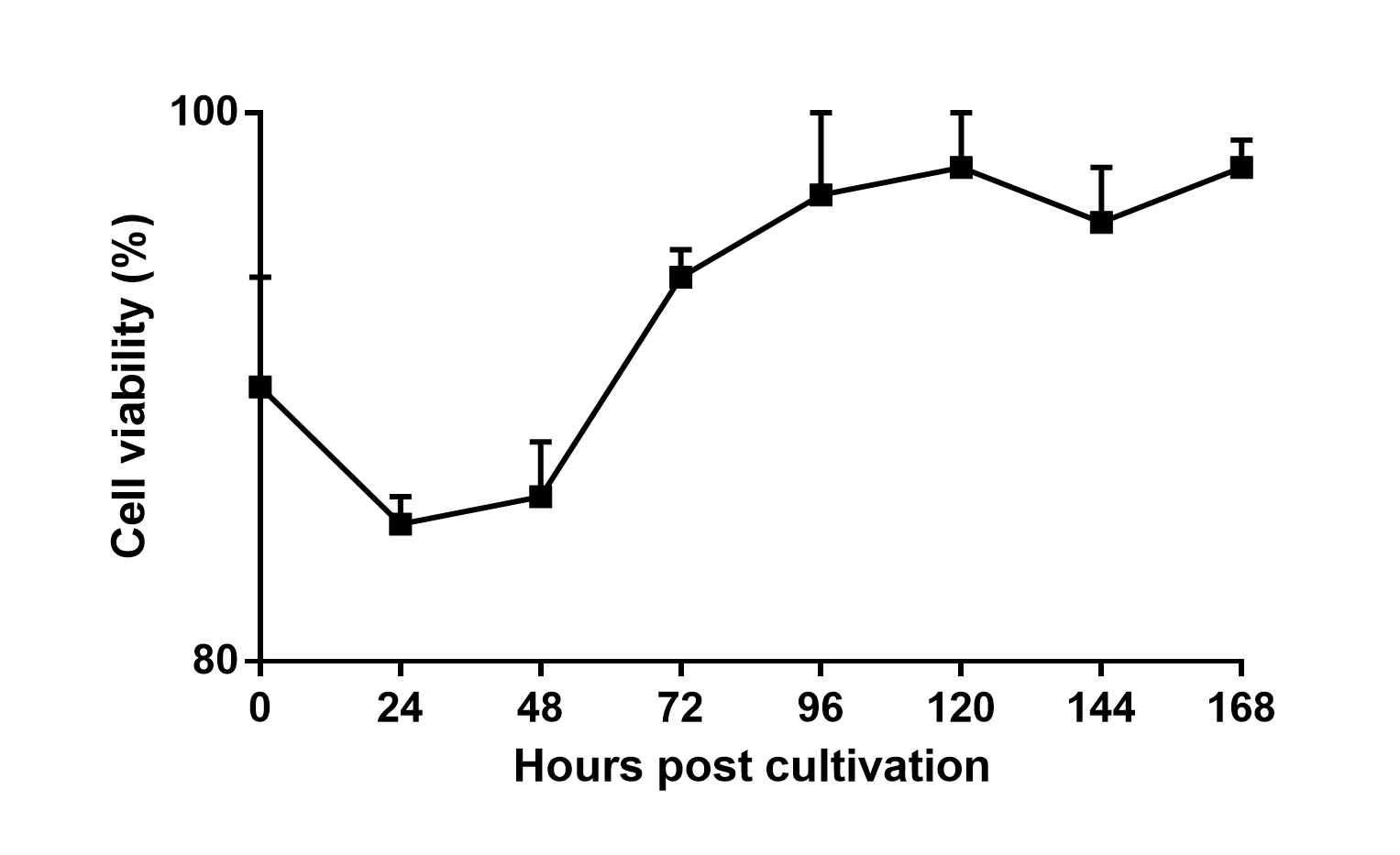

Supplement: S4 Fig — Cell viability was determined by microscopic assessment of cell death upon trypan-blue staining. The average cell viability ± SEM is the result of two independent repeats run in duplicate. (TIF) [file pntd.0007885.s005.tif]
